# Food Neophobia: A Common Challenge Among Brazilian Children with Down Syndrome

**DOI:** 10.3390/nu17071199

**Published:** 2025-03-29

**Authors:** Priscila Claudino De Almeida, Ivana Aragão Lira Vasconcelos, Eduardo Yoshio Nakano, Renata Puppin Zandonadi, Raquel Braz Assunção Botelho

**Affiliations:** 1 Graduate Program in Human Nutrition, University of Brasília, Brasília 70910-900, Brazil; nprialmeida@gmail.com; 2 Department of Nutrition, University of Brasília, Brasília 70910-900, Brazil; renatapz@unb.br (R.P.Z.); raquelbotelho@unb.br (R.B.A.B.); 3 Department of Statistics, University of Brasília, Brasília 70910-900, Brazil; nakano@unb.br

**Keywords:** food neophobia, Down syndrome, prevalence, child, caregiver perception

## Abstract

**Objective:** Food neophobia (FN) is defined as the reluctance to eat new foods. The present study aimed to evaluate FN in Brazilian children with Down syndrome (DS) based on their caregivers’ perceptions. **Method:** This was a descriptive, cross-sectional study. The convenient sample consisted of responses from the caregivers of 231 children aged 4 to 11 years. Recruitment occurred through chain sampling, research, and dissemination via social media profiles, associations, and emails. Caregivers answered sociodemographic questions and the Brazilian Children’s Food Neophobia Questionnaire (BCFNeo), an instrument previously developed and validated for the Brazilian context. Data were exported from the Google Form^®^ platform and analyzed using Excel^®^ and SPSS^®^. Descriptive statistics, the independent Student’s *t*-test, the Mann–Whitney U test, and the Friedman test were used, following the methodology indicated by BCFNeo. **Results:** The results indicated a high FN prevalence rate of 41.1%. The general domain showed the highest prevalence (48.1%). There were no significant differences in FN across age groups (*p* > 0.05), and boys were more neophobic than girls (*p* = 0.006). The school environment emerged as a favorable setting to encourage the consumption of new fruits (*p* < 0.05). **Conclusions:** Children with DS exhibited similar levels of FN compared to Brazilian neurodiverse children. This study highlights the need for further research into eating behaviors in children with DS and emphasizes the school’s role as a space for the promotion of healthy eating habits.

## 1. Introduction

Food neophobia (FN) is common in childhood, particularly between the ages of two and six, and is characterized by strong reluctance or fear regarding trying new foods [1]. This resistance typically occurs during increased independence and autonomy in food choices, but it can also emerge during the first year of life (early neophobia). After the age of six, FN may decrease or disappear; however, it can re-emerge in adulthood or persist into later life. If FN is severe, it may become more pronounced over time [2].

Several individual and environmental factors are associated with FN [3]. Studies have identified these factors in children, including genetic influences; diets or the availability of foods with low variety and nutritional quality; a lack of exposure to new foods; adverse external reactions to new stimuli and social facilitation; pressure and insistence from caregivers to eat; a lack of positive reinforcement or affection during meals; parental influences on the development of eating habits; difficulties in interpreting hunger and satiety cues; and a lack of time to prepare meals. Other factors include sensitivity to the sensory properties of foods; anxiety, personality traits, and a lack of autonomy in children’s eating; a preference for sweet and salty foods; families living in rural areas; low educational levels; and food neophobia in mothers [3,4]. In the general population, FN can lead to a diet with limited variety, but children with special needs, disabilities, or specific health conditions often have even more restricted diets [5].

Among children with disabilities, those with Down syndrome (DS) are a notable group. DS is a genetic condition involving an extra copy of chromosome 21. The three types of DS are trisomy 21, translocation Down syndrome, and mosaic Down syndrome [6]. A range of complications are common in children with DS, including heart defects, gastrointestinal issues, immune disorders, sleep apnea, spine problems, leukemia, dementia [7], hematological anomalies, congenital hypothyroidism [8], obesity [7] or slow weight gain, constipation, gastroesophageal reflux, duodenal atresia, and anorectal atresia/stenosis [8].

The incidence of DS worldwide is approximately 1 in every 1000 births. It is the most common chromosomal condition diagnosed in the United States, affecting 1 in every 640 babies [6]. In Brazil, the estimate is about 1 in every 700 babies [9].

Most individuals with DS are typically fed normally [10], but children may face unique feeding challenges [11]. For instance, complementary feeding generally occurs later than six months of age [12]. These challenges primarily stem from delayed oromotor development, oropharyngeal dysfunction, hypotonia, a small oral cavity, relative macroglossia, reduced jaw strength, and inadequate tongue control, all of which hinder the development of chewing skills [8,12].

Moreover, early surgical or medical interventions can disrupt feeding development and lead to food aversions. Compounding this, caregiver anxiety and stress can negatively affect the feeding process [12,13]. Texture is also a particularly challenging sensory factor regarding food and can influence the feeding behaviors of these children [11,12].

Given these typical feeding difficulties in DS regarding the introduction of food, and considering how early behaviors may shape future eating habits [11], our study hypothesizes that a significant number of children with DS, both boys and girls, will demonstrate high levels of food neophobia, similarly to neurodivergent children, such as those with autism spectrum disorder (ASD) [14].

Research on feeding and nutrition in children with DS is scarce [10]. While FN is widely studied in various groups, particularly in developed countries such as the US and Europe, with the use of diverse assessment tools, no studies have mapped and assessed FN specifically in children with DS [11,15].

Therefore, the objective of this study was to evaluate food neophobia in Brazilian children with Down syndrome, based on the perceptions of their caregivers.

## 2. Materials and Methods

This study adhered to the guidelines of the Declaration of Helsinki [16] and Resolution No. 466 of 12 December 2012 [17]. It was reviewed and approved in advance, and its implementation was authorized by the Research Ethics Committee for Human Beings at the Faculty of Health Sciences, University of Brasília. Approval was granted under registration number 5.438.498 (approved on 30 May 2022). This research is presented according to STROBE [18]. It was a descriptive, cross-sectional study conducted in Brazil.

### 2.1. Participants

This study recruited participants from all federative units of Brazil through snowball recruitment, which was convenient. Caregivers needed to be familiar with their children’s eating and behavioral habits (often following their meals) and consent to participate in the research. Questionnaires where participants stated that they did not wish to participate or those that were filled out incompletely were excluded.

To reach caregivers of children with DS, we explored profiles related to the topic or non-governmental organizations and shared the research via social media platforms (Instagram^®^ [19], Facebook^®^ [20], Twitter^®^, and X^®^), messaging apps, and email to reach the participants. Therefore, dissemination was guided by parent groups/associations/foundations/federations related to DS (such as the Down Syndrome Foundation, Movimento Down [21], the Brazilian Federation of Down Syndrome Associations, and Parents in Motion) and in support of people with DS; contacts with healthcare and education professionals who assisted children with DS; regional councils of nutritionists; and universities or colleges with extension projects (community outreach) that assisted children with DS.

### 2.2. Instruments and Application

As demonstrated in recent research [22], in Brazil, there is only one specific and validated instrument to assess children’s FN [23]. The Brazilian Children’s Food Neophobia Questionnaire (BCFNeo) [23] was chosen, and we used the same methodology and protocol [14,24,25,26] applied in Brazil to make the data comparable and unify the method of evaluation.

The first section of the instrument addressed the sociodemographic aspects and health conditions of the caregiver/child, with questions related to: (1) the caregiver responsible for the child; (2) sex; (3) age; (4) family income; (5) place of residence; (6) the number of residents in the same household; and (7) other medical diagnoses or health conditions, such as dietary restrictions.

The second section was represented by the complete instrument (BCFNeoTot), with 25 items used to evaluate food neophobia in specific contexts and environments. For each item, there were 5 response options. However, to calculate the score, the order of the responses was reversed, and they were coded from 0 to 4, so, the higher the score, the greater the FN. The total number of items was divided into three food neophobia domains (possible score from 0 to 100 points): general food neophobia (FNgen), with 9 items and a possible score from 0 to 36, and food neophobia for fruits (FNfru) and food neophobia for vegetables (FNveg), with 8 items each and a possible score from 0 to 32.

Based on the sum of the points from all domains (for the BCFNeoTot), FN was classified according to its degree: low (up to 40 points), moderate (from 41 to 65 points), and high (from 66 points onwards). On the other hand, within each domain, FN was low up to 13 points, moderate from 14 to 21 points, and high from 22 points onwards. For the analysis comparing the children’s possible behavior in different environments, the mean scores (in this case, the score ranges from 0 to 4) of nine specific items of the instrument were calculated. These items assessed the caregiver’s perceptions of the child’s eating behavior in different environments (home, school, or a friend’s house) to determine if the environment influenced their FN.

For the family income variable, financial values were converted based on the exchange rate of USD 1 to BRL 5.80, i.e., the rate on 1 November 2024 [27]. With this conversion rate, the minimum wage (MW) for analysis was BRL 1412.00 [28], i.e., approximately USD 245.00.

The BCFNeo and its informed consent form were available through the online platform Google Forms^®^. Data collection occurred from August 2022 to November 2024.

### 2.3. Statistical Analysis

After importing the data from the Google Forms^®^ platform, the analysis was conducted using SPSS^®^ 20.0 and Excel^®^. Descriptive statistics were used, including the mean and standard deviation or frequencies and percentages, depending on the nature of the variable. The analysis was divided into two age groups (4–7 and 8–11 years) and two sexes (female and male). The mean FN scores (overall and for each domain) were compared between the sex and age groups of the children using the independent Student’s *t*-test. Frequencies were also categorized according to three ordinal levels, and the Mann–Whitney U test was used to compare neophobia levels between sex and age groups. The Friedman test, followed by the Wilcoxon test with Bonferroni correction, was performed to analyze differences in the FN scores by environment, sex, and age. The normality of the observations was tested using the Kolmogorov–Smirnov test with Lilliefors correction. All tests considered two-tailed hypotheses and a significance level of 5%.

## 3. Results

We received 254 responses to the instrument. However, 23 questionnaires had to be excluded: 19 corresponded to children not within the study’s age range, and four corresponded to children without a diagnosis of DS. All questionnaires were fully completed, and all respondents consented to participate in this research. Therefore, the final sample consisted of the caregivers of 231 Brazilian children with DS.

The caregivers were primarily female (n = 218; 94.4%), mostly mothers (n = 215; 93.1%), living in urban areas (n = 220; 95.2%), married or in a stable union (n = 183; 79.2%), and postgraduates (n = 62; 26.8%). The mean age of the caregivers was 41.7 ± 8.0 years, and the mean number of people living in the same house was 3.8 ± 0.9, with a maximum of seven people. The majority (n = 133; 57.6%) reported a monthly family income below 8190.00 (Supplementary Table S1).

The sample was well balanced (Table 1); the children were mostly boys (n = 125; 54.1%), and the mean age was 6.8 ± 2.2. As for medical diagnoses, they mostly had only DS (n = 168; 72.7%). The others (n = 63; 27.3%) could have one or more diagnoses.

The prevalence (BCFNeoTot) of high and moderate FN was 41.1% and 32.9%, respectively (Table 2). Considering the high FN, the domain with the highest prevalence was FNgen (48.1%), and FNfru was the one with the lowest prevalence of high FN (37.2%).

There was no difference in FN when comparing ages (*p* > 0.05). For all domains (FNgen, FNfru, and FNveg) and overall (BCFNeoTot), sex indicated a difference; boys were more food neophobic (Table 3).

Table 4 shows the FN for fruits and vegetables and the FN with a friend’s influence, in three different environments (school, home, a friend’s home), without considering the domains, and using specific items. When comparing the three environments, the school was where the children, in total, had lower FN for fruits (*p* < 0.001). The friend’s home environment was where the children, in total, had higher FN for vegetables (*p* = 0.001) and higher FN with a friend’s influence (*p* = 0.004). The environment impacted FN according to sex. Girls and boys were less neophobic at school and more neophobic at friends’ houses, except for boys regarding FN under a friend’s influence, who maintained similar averages in all environments (*p* = 0.134). Considering the age groups, younger children displayed lower neophobia toward fruits at school (*p* < 0.001) but showed higher neophobia for vegetables when influenced by friends at a friend’s house (*p* = 0.002). Among older children, fruit neophobia at school was significantly lower than fruit neophobia at a friend’s house (*p* = 0.05).

## 4. Discussion

The profile of the respondents in the present study was similar to that observed in other research, requiring caregivers’ knowledge of children’s diets to measure FN [14,24,25]. As no studies were found evaluating FN in children with DS, we compared our results with those of a study on Brazilian children who were mostly neurodiverse [24], as well as a study on predominantly neurotypical children in the federal district (FD) (Brazil) [25] and one on other Brazilian children diagnosed with ASD [14], all within the same age group and using the same instrument. When comparing the prevalence of high and moderate FN in children with DS (41.1% and 32.9%, respectively), although these rates were considered significant, we observed that the profile was similar to that of predominantly neurotypical Brazilian children (33.4% and 36.7%) [24] and children from the FD (42.9% and 32.6%) [25] than to that of children with ASD (73.9% and 18.0%) [14].

Another point is that the caregivers may have underestimated the refusal of new foods in the instrument’s questions due to being more accustomed to the children’s feeding difficulties, having different expectations regarding the child’s behavior and abilities, and considering certain reactions as normal. A study indicates that the caregivers of young children with DS do not perceive more feeding problems than the parents of typically developing children. When asked about feeding issues, they tend to underreport them because they operate within a different framework of expectation for children with DS [29].

Although neophobia had a high prevalence, similar to the perceptions of parents of neurodiverse children, it is noteworthy that high food neophobia in children with DS was more pronounced in the general domain compared to the domains of fruits and vegetables. This trend was less evident in the other groups of children from the other studies, with the FD study showing greater divergence specifically in the vegetable domain [25].

A study in Poland aimed to assess the diets of children with DS and to identify potential dietary errors made by parents, using 211 participants. The mean age of the study group with DS was 6.6 years ± 5.8 years (min 0.5 years–max 30 years; median 4.5 years). Vegetables and fruits were consumed with a frequency of once a day, as indicated by their parents, totaling 83 (42.6%) and 87 (44.6%), respectively. However, extremes were observed. Parents indicated that their children did not eat vegetables (n = 4; 2.1%) and fruits (n = 3; 1.5%). Regarding other food groups, there was greater refusal. Parents reported that their children did not eat dairy products at all (n = 36; 20%), as well as eggs (n = 22; 11.3%) and fish (n = 13; 6.7%). They did not give sweets to their children at all (n = 64; 32.8%), as well as nuts for health reasons (allergy) (n = 24; 12.3%) and nuts without justification (n = 141; 72.3%). The authors concluded that most DS subjects were usually well fed, but their parents made dietary mistakes. For example, when no absolute indications exist, food groups should not be excluded [10]. This may help to explain the higher prevalence of neophobia observed in the general dietary domain.

Regarding age, it was anticipated that globalization would lead to less variation in dietary patterns across countries and, generally, that FN would decline as children grow older [15]. However, this study involving children with DS found no significant decrease in FN with advancing age (*p* > 0.05). Similar findings have been reported in neurodiverse Brazilian children [24,25] and those with ASD [14].

When comparing different studies within the same country, i.e., Brazil, two studies conducted with the caregivers of mostly neurotypical children showed that boys could be more neophobic [24,25]. Our study had results that were similar to two studies with neurodiverse children, indicating that boys with DS are more neophobic than girls. On the other hand, a study with Brazilian children with ASD [14] found no distinction in the prevalence or level of FN between the sexes. An explanation may be that girls like fruits and vegetables more than boys. Boys tend to like foods that are high in fat and sugar, meat, meat products, and eggs more than girls, as shown in a cross-sectional survey that examined age and gender differences in the food preferences of British schoolchildren aged 4 to 16 years old (n = 1291) [30]. Children’s innate preferences for sweet and salty flavors and foods rich in fats and/or sugars are factors that can influence FN [4].

A study on the eating and lifestyle habits of DS children (n = 34) aged two to sixteen years old, attending a multispecialist program to identify their challenges, showed that 53% of the families had received no nutrition counseling when entering the program, and 47% relied on different sources, including the Internet. Due to the lack of adequate knowledge about food and nutrition, parents believed that, due to the eating patterns of their children with DS, it was important to supplement the child. Given this, nutritional excesses could occur. When a multidisciplinary team monitored children with DS, their eating habits and lifestyles were analyzed, and the treatment was well directed, many benefits arose. Anthropometrically, the risk of overweight/obesity can be curtailed in DS [31]. It is essential to educate the caregivers of children with DS so that they can feed their children appropriately; this can improve their health and well-being [10].

Analyzing the environments, school proved to be one of the primary environments in which children exhibited less food neophobia toward fruits (1.97 ± 1.25). This is not necessarily due to the influence of friends in the school environment (2.06 ± 1.19). School stands out as an environment where children are less likely to present FN toward fruits, as observed in the federal district [25]. The National School Feeding Program (PNAE) offers school meals and food and nutritional education activities to students at all stages of public basic education [32]. It is a right of students in public basic education and a duty of the state. In addition to food and nutrition, the program aims to form healthy eating habits [33]. Private schools have their own rules and legislation, but they must have nutritionists and develop food and nutritional education projects. If they wish, they can follow the PNAE [34]. Food and nutritional education and the presence of a nutritionist in schools may be the reasons that this environment has lower levels of neophobia.

Schools, as key spaces for the social inclusion of these children, also stand out as promising environments in encouraging the exploration and consumption of new foods, according to the study’s findings. By integrating nutrition and food-related topics into the school curriculum through ongoing, cross-disciplinary projects, such as cooking workshops or interactive lessons, schools can foster curiosity and a willingness to try new foods. These efforts, sustained over time, have the potential to significantly reduce food neophobia and promote healthier eating habits in the long term. Eating habits are intertwined with neurodevelopmental evolution; weaning is a pediatric strategic knot. Progress in weaning is not easy, because chewing can be critical. Parents may struggle with food consistency for several years. However, it can be managed: families who entered a support program when their children were at a young age and were followed for a long time experienced fewer problems [31]. Children with DS are diagnosed early in life and referred to multidisciplinary teams. This may be the reason that the prevalence of high FN in children with DS is lower than in children in the FD with ASD.

This study presents some limitations, like the snowball sampling (SS) method used to disseminate the research and the limitations of convenience sampling. However, due to the particularities of the sample, as is the case in children with DS, living in a country with a large territorial area, such as Brazil, this strategy helps to obtain a larger sample. The snowball sampling method is appropriate when the target population is difficult to access (a hidden population), when the study may involve sensitive issues, and when compiling a population list for random selection poses a challenge [35]. Convenience sampling is beneficial since there are no national data on prevalence in the country and its distribution among states [36]. Data found in the literature indicate the number of births [37,38]. Other studies that have evaluated FN in Brazilian children used the same methodology, such as those that have evaluated FN in children with ASD [14,24,25,26].

Another potential limitation of this study relates to the instrument used to measure FN. It involves an indirect approach, where the caregiver’s responses are based on their memory and perceptions of how they believe the child would behave in each situation or context, rather than directly assessing the child’s behavior [39]. Future research should analyze FN at school to explain the factors that make this the environment in which children have lower FN, and to explain the factors involved in the observed differences between the sexes. There are other strategies for data collection that could complement, reduce, or eliminate this bias, such as observational prospective studies. Alternatively, along with scale data, a complementary dietary assessment questionnaire, such as a food diary or food frequency questionnaire, could be applied to provide more insight into children’s food consumption [39].

The prospective study by Nicklaus et al., 2005 [40], for example, used behavioral measures of food choices through the observation of the lunches of French children under 4 years old and later, when they were older, to assess early food variety seeking and follow up over time to relate it to potential FN. However, as an observational study with follow-up, it had a relatively small sample size (n = 339) given the duration of the data collection period (the first phase lasted 17 years), greater difficulty in operationalization, and consequently higher costs [39,40].

The instrument used in our research, the BCFNeo [23], was developed based on three existing instruments, including the FNS [1], with additional questions created by the researchers. Following an extensive literature review, the Delphi method was employed for internal validation (content validation and semantic evaluation). The instrument, comprising 25 items, was tested with a sample of children’s caregivers to assess the internal consistency and reproducibility [23].

It is also worth noting that the BCFNeo was validated with the participation of neurodiverse children, making it representative of Brazil. During its validation, children with ASD (n = 33; 2.96%), DS (n = 23; 2.06%), and other diagnoses participated among the 1112 children [24].

Child caregivers must introduce new foods gradually and without pressure, regardless of the child’s condition (neurodivergent or neurotypical). Always emphasizing the importance of food and its varied consumption for health, and offering a variety of healthy foods from an early age, can help to reduce the resistance to new flavors and textures. In addition, creating a positive, distraction-free mealtime environment can encourage children to try new foods. It is important not to force children to eat but rather to encourage curiosity and food exploration. In addition to caregivers, the importance of health professionals in overcoming food neophobia is emphasized. Health professionals should educate parents about the importance of a diverse diet and provide strategies for the introduction of new foods, as well as monitoring the child’s progress and adjusting their approaches as necessary. Interventions such as involving children in food preparation at home and even planting, exploring, smelling, and tasting new spices can be useful to engage children in the food universe, increasing their acceptance and reducing food neophobia.

Furthermore, DS remains an underexplored condition in the scientific literature, with limited research addressing nutrition and eating behaviors in this population. It is crucial to assess and measure the degree of FN in children with DS to provide personalized guidance to parents regarding their children’s eating behaviors. From a public policy perspective, such assessments can help to map the extent of the issue among Brazilian children, enabling the qualification and training of professionals and multidisciplinary teams. This information can guide the development of specific recommendations, updates in management practices, and initiatives to promote the health and well-being of these children and their families.

## 5. Conclusions

To our knowledge, this is the first study that has assessed and measured FN in children with DS. The results allow us to conclude that Brazilian children with DS exhibit similar levels of FN to neurodiverse Brazilian children but are less neophobic compared to children with other types of neurodivergence, such as children with ASD. This finding raises questions about whether the age at diagnosis, differentiated eating issues, and social interactions during meals influence FN levels, depending on the type of neurodivergence.

## Figures and Tables

**Table 1:** Sociodemographic data and children’s profile (n = 231).

	Category	Sample	
		N	%
Sex	Female	106	45.9
	Male	125	54.1
Age	4 years old	46	19.9
	5 years old	38	16.5
	6 years old	32	13.9
	7 years old	26	11.3
	8 years old	29	12.6
	9 years old	27	11.7
	10 years old	13	5.6
	11 years old	20	8.7
Diagnoses *	Only Down’s syndrome	168	72.7
	Food intolerance	31	13.4
	Food allergies	20	8.7
	Autism spectrum disorder	8	3.5
	Heart disease	5	2.2
	Thyroid diseases **	3	1.3
	Celiac disease	2	0.9
	Eating disorders ***	2	0.9
	Cerebral palsy, brain injury, or hydrocephalus	2	0.9
	Others diagnoses ****	5	2.2

* Children may have one or more diagnoses. ** Hypothyroidism, hyperthyroidism, or Hashimoto’s thyroiditis. *** Anorexia/bulimia. **** Such as other syndromes, respiratory diseases, and others.

**Table 2:** Distribution of the sample according to food neophobia classification (n = 231), Brazil.

	Food Neophobia		
	Low n (%)	Moderate n (%)	High n (%)
Domain of neophobia in general * (FNgen)	62 (26.8%)	58 (25.1%)	111 (48.1%)
Domain of neophobia for fruits * (FNfru)	72 (31.2%)	73 (31.6%)	86 (37.2%)
Domain of neophobia for vegetables * (FNveg)	63 (27.3%)	67 (29.0%)	101 (43.7%)
TOTAL INSTRUMENT SCORE ** (BCFNeoTot)	60 (26.0%)	76 (32.9%)	95 (41.1%)

* Domain score cutoff points: low (up to 13 points); moderate (14 to 21 points); high (22 points or more). ** Total score cutoff points: low (up to 40 points); moderate (41 to 65 points); high (66 points or more).

**Table 3:** Food neophobia scores and classification distribution by sex and age group (n = 231).

	Sex			Age		
	Girls (n = 106)	Boys (n = 125)	*p*-Value	4–7 y (n = 142)	8–11 y (n = 89)	*p*-Value
General neophobia (FNgen)						
Score; Mean (SD)	17.92 (8.64)	21.18 (9.36)	0.007 *	19.73 (8.86)	19.61 (9.68)	0.924 *
Distribution; n (%)						
Low (up to 13)	33 (31.1%)	29 (23.2%)		38 (26.8%)	24 (27.0%)	
Moderate (14 to 21)	34 (32.1%)	24 (19.2%)	0.006 **	35 (24.6%)	23 (25.8%)	0.876 **
High (22 or more)	39 (36.8%)	72 (57.6%)		69 (48.6%)	42 (47.2%)	
Fruit neophobia (FNfru)						
Score; Mean (SD)	16.41 (8.49)	19.27 (9.19)	0.015 *	18.18 (8.94)	17.6 (9.06)	0.629 *
Distribution; n (%)						
Low (up to 13)	40 (37.7%)	32 (25.6%)		41 (28.9%)	31 (34.8%)	
Moderate (14 to 21)	37 (34.9%)	36 (28.8%)	0.005 **	47 (33.1%)	26 (29.2%)	0.483 **
High (22 or more)	29 (27.4%)	57 (45.6%)		54 (38.0%)	32 (36.0%)	
Vegetable neophobia (FNveg)						
Score; Mean (SD)	16.96 (8.67)	19.85 (9.00)	0.014 *	18.98 (8.84)	17.8 (9.11)	0.330 *
Distribution; n (%)						
Low (up to 13)	35 (33.0%)	28 (22.4%)		35 (24.6%)	28 (31.5%)	
Moderate (14 to 21)	31 (29.2%)	36 (28.8%)	0.049 **	45 (31.7%)	22 (24.7%)	0.611 **
High (22 or more)	40 (37.7%)	61 (48.8%)		62 (43.7%)	39 (43.8%)	
TOTAL (BCFNeoTot)						
Score; Mean (SD)	51.28 (23.70)	60.3 (25.75)	0.006 *	56.89 (24.94)	55 (25.67)	0.580 *
Distribution; n (%)						
Low (up to 40)	32 (30.2%)	28 (22.4%)		37 (26.1%)	23 (25.8%)	
Moderate (41 to 65)	40 (37.7%)	36 (28.8%)	0.018 **	47 (33.1%)	29 (32.6%)	0.928 **
High (66 or more)	34 (32.1%)	61 (48.8%)		58 (40.8%)	37 (41.6%)	

* Independent Student *t*-test; ** Mann–Whitney U test.

**Table 4:** Scores and distribution of food neophobia according to the environment by age group and sex (n = 231).

		School Mean (SD)	Home Mean (SD)	Friend’s Home Mean (SD)	*p* *
	Total	1.97 (1.25) ^a^	2.14 (1.26) ^b^	2.19 (1.23) ^b^	<0.001
	Girls	1.77 (1.20) ^a^	1.96 (1.24) ^ab^	2.00 (1.16) ^b^	0.001
Fruit neophobia ^1^	Boys	2.14 (1.27) ^a^	2.30 (1.26) ^ab^	2.34 (1.26) ^b^	0.005
	4–7 y	1.99 (1.24) ^a^	2.18 (1.22) ^b^	2.23 (1.22) ^b^	<0.001
	8–11 y	1.93 (1.27) ^a^	2.08 (1.33) ^ab^	2.11 (1.24) ^b^	0.050
	Total	2.19 (1.18) ^a^	2.20 (1.23) ^a^	2.35 (1.19) ^b^	0.001
	Girls	2.03 (1.14) ^a^	1.92 (1.14) ^a^	2.23 (1.14) ^b^	0.002
Vegetable neophobia ^2^	Boys	2.32 (1.20) ^a^	2.45 (1.25) ^ab^	2.45 (1.22) ^b^	0.045
	4–7 y	2.19 (1.17) ^a^	2.29 (1.21) ^ab^	2.37 (1.16) ^b^	0.002
	8–11 y	2.18 (1.20) ^a^	2.07 (1.26) ^a^	2.30 (1.24) ^a^	0.174
	Total	2.06 (1.19) ^a^	2.06 (1.19) ^a^	2.19 (1.14) ^b^	0.004
	Girls	1.90 (1.14) ^a^	1.92 (1.13) ^a^	2.06 (1.11) ^b^	0.022
Neophobia with friend’s influence ^3^	Boys	2.19 (1.22) ^a^	2.19 (1.22) ^a^	2.30 (1.15) ^a^	0.134
	4–7 y	2.06 (1.14) ^a^	2.05 (1.16) ^a^	2.23 (1.09) ^b^	0.002
	8–11 y	2.06 (1.26) ^a^	2.09 (1.24) ^a^	2.12 (1.20) ^a^	0.564

^1^ At school, at home, or at a friend’s house: the taste of a new fruit; ^2^ at school, at home, or at a friend’s house: the taste of a new vegetable; ^3^ at school, at home, or at a friend’s house: a friend’s acceptance would lead the child to taste the food. * Friedman test followed by Wilcoxon post hoc test with Bonferroni correction. For the post hoc tests, environments with the same letters do not differ significantly.

## Data Availability

The original contributions presented in this study are included in the article and Supplementary Materials. Further inquiries can be directed to the corresponding author.

## Supplementary Materials

The following supporting information can be downloaded at https://www.mdpi.com/article/10.3390/nu17071199/s1, Table S1: Characterization of caregivers and their children (n = 231).
