# Supplementary material for: Food Neophobia: A Common Challenge Among Brazilian Children with Down Syndrome
Source: Nutrients. 2025 Mar 29;17(7):1199. doi: 10.3390/nu17071199 (PMC11990528; doi:10.3390/nu17071199)
Supplement: Supplementary file 1 [file nutrients-17-01199-s001.zip › nutrients-3553980-supplementary.pdf]

Table S1. Characterization of caregivers and their children (n=231).

	Categories	Sample	
		**n**	**%**
**Caregivers Sex**	Female	218	94.4%
	Male	13	5.6%
**Caregivers' degree of kinship**	Mothers	215	93.1%
	Fathers	12	5.2%
	Grandparents	3	1.3%
	Sisters or brothers	1	0.4%
**Caregiver Marital status**	Single	29	12.6%
	Married/ stable union	183	79.2%
	Separate/ divorced	19	8.2%
**Educational Level of Caregivers**	From 1st to 4th grade of elementary school (former primary)	1	0.4%
	From 5th to 8th grade of elementary school (former gym)	10	4.3%
	High School (2nd degree) incomplete	13	5.6%
	Complete high school	42	18.2%
	Higher Education Incomplete	24	10.4%
	Higher Education Complete	59	25.5%
	Postgraduate	62	26.8%
	Master's degree	10	4.3%
	Doctorate degree	7	3.0%
	Postdoctoral	3	1.3%
**Housing area**	Urban area	220	95.2%
	Rural area	11	4.8%
**Number of Residents in the Same House**	Two	17	7.4%
	Three	55	23.8%
	Four	118	51.1%
	Five	35	15.2%
	Six	5	2.2%
	Seven	1	0.4%
**Monthly family income**	No income	1	0.4%
	Up to 1 MW	35	15.2%
	Up to 2 MW	34	14.7%
	Up to 3 MW	28	12.1%
	Up to 4 MW	18	7.8%
	Up to 5 MW	18	7.8%
	Up to 6 MW	12	5.2%
	Up to 7 MW	9	3.9%
	Up to 8 MW	7	3.0%
	Up to 9 MW	8	3.5%
	Between 10 and 12 MW	16	6.9%
	Between 13 and 15 MW	9	3.9%
	Above 15 MW	20	8.7%
	Not informed	16	6.9%
**Caregivers age**	Up to 20 y/o	2	0.9%
	Between 21 and 30 y/o	17	7.4%
	Between 31 and 40 y/o	72	31.2%
	Between 41 and 50 y/o	119	51.5%
	Between 51 and 60 y/o	21	9.1%
